# Does Sex Trade with Violence among Genotypes in *Drosophila melanogaster*?

**DOI:** 10.1371/journal.pone.0001986

**Published:** 2008-04-16

**Authors:** Larry G. Cabral, Brad R. Foley, Sergey V. Nuzhdin

**Affiliations:** 1 Department of Biological Sciences, California State University Sacramento, Sacramento, California, United States of America; 2 Evolution and Ecology, University of California Davis, Davis, California, United States of America; 3 Molecular and Computational Biology, University of Southern California, Los Angeles, California, United States of America; University of Exeter, United Kingdom

## Abstract

The evolutionary forces shaping the ability to win competitive interactions, such as aggressive encounters, are still poorly understood. Given a fitness advantage for competitive success, variance in aggressive and sexual display traits should be depleted, but a great deal of variation in these traits is consistently found. While life history tradeoffs have been commonly cited as a mechanism for the maintenance of variation, the variability of competing strategies of conspecifics may mean there is no single optimum strategy. We measured the genetically determined outcomes of aggressive interactions, and the resulting effects on mating success, in a panel of diverse inbred lines representing both natural variation and artificially selected genotypes. Males of one genotype which consistently lost territorial encounters with other genotypes were nonetheless successful against males that were artificially selected for supernormal aggression and dominated all other lines. Intransitive patterns of territorial success could maintain variation in aggressive strategies if there is a preference for territorial males. Territorial success was not always associated with male mating success however and females preferred ‘winners’ among some male genotypes, and ‘losers’ among other male genotypes. This suggests that studying behaviour from the perspective of population means may provide limited evolutionary and genetic insight. Overall patterns of competitive success among males and mating transactions between the sexes are consistent with mechanisms proposed for the maintenance of genetic variation due to nonlinear outcomes of competitive interactions.

## Introduction

The mechanisms underlying the maintenance of genetic variation in fitness traits are poorly understood. Territorial interactions, for instance, require an investment of energy and time. In homogenous environments, when holding a territory results in higher mating success, more aggressive males might be expected to take over the population. Otherwise, if mating success is not related to territorial success, genotypes resulting in males averse to aggression would be expected to spread as these males invest less in expensive fights. Even under constant lab conditions, however, a great deal of additive genetic variation is maintained in aggressive traits over many generations [1], [2]. In sexual signalling as well, recent work on the ‘lek paradox’ has focussed on the theoretical expectation that variation in the direction of selection should be depleted such that the effort spent in signalling seems paradoxical [3], [4]. In several systems, active mechanisms (as opposed to passive mechanisms, such as mutation-selection balance) that might explain the maintenance of this kind of variation have been demonstrated. While the details of these mechanisms are diverse, many of them imply that environments that include other individuals are never in fact homogeneous – as interacting partners might themselves vary [5], [6]. The effects of intergenomic interactions on the relative fitness of individuals are often called Indirect Genetic Effects (IGEs).

Given that interactions with other individuals are an important component of fitness, there may be more than one optimal behaviour depending on the behavioural predisposition of others in a population. Competitive mating interactions are one example of how IGEs can affect fitness in ways that may help explain maintenance of variation [7]. Success in competitive interactions for individuals with different genotypes may be intransitive between competing types [8], frequency dependant [9], or dependent on variation in the preferences of potential mates [10]–[12]. In each of these cases, the Evolutionarily Stable Strategy (ESS) will be a mix of strategies among individuals seeking mates. Theoretical work suggests that even in homogenous environments, variation in IGEs may not be lost [7]. While variants with a consistent advantage are likely to go to fixation quickly, variants with intransitive patterns of success against different competitors are likely to be retained in populations. To establish the importance of these mechanisms in the maintenance of genetic variation, however, it is necessary to demonstrate that the behaviour and its outcome are not properties of a genotype, but rather emerge from interactions between individuals of specific genotypes. Although a few such systems have been well described, genetically-driven analyses are practically nonexistent.

The side-blotched lizard is one of the best genetically and behaviourally characterised systems of maintenance of behavioural variation due to intransitive success due to IGEs [8]. In this system, conveniently, behavioural strategies correlate with three distinct, genetically determined colour morphs. Stable polymorphism in behavioural strategy is maintained because no behavioural type is able to win more matings than the other two types, and mating success of a morph follows a rock-scissors-paper dynamic. Loosely, aggressive orange males win when competing with the pair-bonding blue morph. The blue morph is able to fend off the sneaky yellow morph, but the yellow morph can steal matings when competing against orange males. No single morph can go to fixation in a large population, because as it increases in frequency, the relative fitness of its dominating competitor likewise increases and polymorphism is maintained.

While such distinct colour and behavioural morphs are not evidently ubiquitous in animals, more subtle tradeoffs between mating and aggressive strategies may exist in other groups, such as Drosophila. Studies, including those in naturalistic settings [13], have shown a large amount of genetic diversity in Drosophila behaviours. In particular, Drosophila have been shown to be an interesting group in which to analyze aggressive and territorial behaviour [1], [14], [15]. There is a vast amount of genetic resources readily available in *D. melanogaster* as well as physiological, neurological and metabolic information [16], making this an attractive system for studying the evolution of behavioural strategies and the maintenance of genetic variation due to IGEs [17].

Territorial success contributes to mating success in *Drosophila melanogaster* [18]. An overall female preference for territorial males has been noted among mated females, although in virgin females the direction of preference for territorial males apparently varies among populations. Additionally, Hoffmann and Cacoyianni have found that males selected for increased aggression had a net advantage in territorial contests; but as the ratio of territorial to non-territorial males increased, this advantage was reversed. While they looked at broad patterns within and across populations, Hoffmann and Cacoyianni did not focus on interactions between individuals of specific genotypes within populations. Some more recent studies [1], [15] have also been primarily concerned with population-level analyses among selected populations of behaviours in relation to territorial success. Conversely other studies that focussed on interactions among individuals, have only been conducted on single genotypes [14], [19], and have not considered the dynamics of interactions between individuals of different genotypes.

Here, we have studied the genetics of the variation in outcomes of territorial interactions between males, and, in the context of the outcomes of these interactions, the relative mating success of these males with females of different genotypes. Panels of inbred *D. melanogaster* lines are commonly used to study the genetics of phenotypic traits, and may be employed in behavioural analyses [17]. This facilitates the analyses of pairwise and higher order IGEs in a way impossible with randomly selected members of an outbred population. Using inbred lines derived from a natural population, we tested several hypotheses related to the maintenance of variation in aggressive traits in this species.

First, we were curious to see whether territorial success in males is a transitive trait, where, as has been suggested, the more initially aggressive male normally wins a territorial interaction [14]. If mean aggression varies quantitatively, and there is a linear relation between aggression and territorial success, we would expect a hierarchy of territorial success among the panel of lines. Alternatively, intransitive interactions may be important, similar to the rock-scissors-paper model of side-blotched lizards [8]. Second, we employed artificially selected lab stocks with behaviourally extreme phenotypes, and tested them against the naturally derived lines, in order to determine whether there are tradeoffs associated with these extreme behaviours which would explain why they are not common in nature. Intransitive patterns of territorial success might be one expected tradeoff, if extreme behavioural types are not competent against all other behavioural strategies in the population, as predicted in [7]. If there is a direct fitness benefit to territorial success, intransitive patterns of success between genotypes could maintain variation in aggressive strategies. Third, we wished to assay genetic variation in virgin female choice across a diversity of male genotypes and interaction outcomes. Different female preferences for territorial traits, or differential investment by males in territoriality as opposed to other sexual signals might be expected to maintain genetic diversity in aggression, as has been demonstrated for maintenance of colour polymorphism in guppies [10].

## Results

### Experiment 1: aggression assays

Aggression trials between male genotypes were in a standard dyadic format in an enclosed arena with a single food source. We were interested in determining whether some lines were consistently more aggressive than others, and whether territorial success is transitive among genotypes. Eight nearly-isogenic lines derived from a natural population (Winters California) were used. Males that held and defended the food source from approaches by the other male were termed territorially successful. We assume that the relative frequency of wins in some way reflects underlying behavioural variation. As aggressive males have been shown to be more successful in winning and holding territories [14], territorial success was taken to be an indicator of aggressive tendencies. Past work on aggression and territoriality in *D. melanogaster* has described a great deal of genetic variation in the outcomes of aggressive interactions [15].

We ran our aggression trials in two blocks: the pattern of territorial success within the eight natural Winters lines (see Materials and Methods) did not vary significantly between the two blocks (n = 532, df = 7, χ^2^ = 9.2, P = 0.238) therefore the results for the Winters lines were combined. Within the Winters lines, there was a very strong, linear rank order among lines for success in aggressive encounters (n = 532, DF = 7, χ^2^ = 38.947, P<0.0001) (Table 1). Overall levels of territorial success are a good predictor of the outcome of individual interactions in all cases (i.e. interactions are transitive) when considering the Winters inbred lines competing only among themselves. In only one interaction (between Winters lines 145 and 75) was there any indication that the results might be different from those expected from relative performance against the other genotypes.

**Table 1:** Territorial success scores for paired combinations of Winters inbred lines of *D. melanogaster*.

	Competing Lines								
Focal Line	W145	W75	W134	W89	W17	W58	W83	W23	Focal Total
W145		4*	14	13	14	16	17	14	**92** ***
W75	0.017		9	11	15	15	14	13	**92** ***
W134	0.145	0.630		12	11	11	13	14	76
W89	0.462	1.000	0.466		12	9	11	16	69
W17	0.328	0.206	1.000	0.622		10	11	13	58
W58	0.074	0.205	1.000	0.625	1.000		13	10	57
W83	0.061	0.610	0.622	1.000	1.000	0.321		12	**47** **
W23	0.794	1.000	0.603	0.114	0.457	0.801	0.612		**41** ***

Scores are counts of wins for males of the focal line after 19 trials.

* P<0.05

** P<0.01

*** P<0.0001

Scores that maintain significance under a sequential Bonferroni correction for multiple testing are indicated in bold.

In Block 2 we also analysed lines artificially selected for heightened aggressive behaviours (Agg) and the unselected control lines (Neut) from a previous experiment [1] that were kindly provided to us by Dr. Greenspan (see Materials and Methods for detail). The Agg and Neut lines provided us with an opportunity to examine the patterns of behaviour for highly aggressive or unselected (but lab adapted) strategies respectively against the natural variation represented by the Winters lines. Their pattern of success was evaluated separately against the Winters lines, and each other. Considering Block 2 alone, there was a similarly strong differentiation among lines in aggression, with the selected Agg line topping the hierarchy, and the unselected Neut line showing the least aggression (n = 405, DF = 9, χ^2^ = 40.654, P<0.0001). Individual tests for significance of aggressive wins show that, for the most aggressive or non-aggressive lines, the proportion of trials won or lost are much more extreme than would be expected if territorial success were determined by chance (i.e. assuming an equal probability of success for either line). The significance of the most extreme values holds after sequential Bonferroni correction.

**Figure 1:**
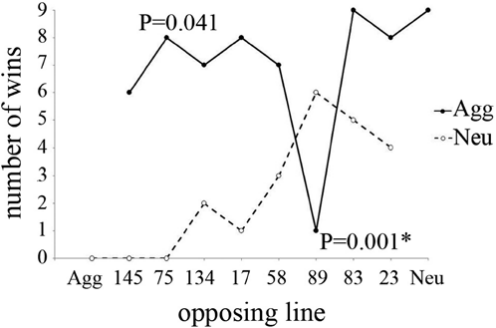
The number of wins of the Aggressive and Neutral lines when competed against the Winters inbred strains. Winters lines are ordered by mean level of territorial success from most to least successful. * significant under sequential Bonferroni for multiple testing

When competing the selected Agg and Neut lines against the Winters lines, two interactions were significantly different from those we expected, given the results in the other trials (Figure 1). Despite generally winning few territorial interactions against most other genotypes, males from Winters line 89 were the only ones to win more than 3 trials against the selected Agg line. A two-tailed, Fisher's Exact Test indicated that the probability of such a result is highly unlikely to be due to chance (n = 9, P = 0.0013) and, indeed, this result maintained significance under a Bonferroni correction for 17 tests. Similarly, the Agg line won more often than expected against Winters line 75 given their success in trials with other genotypes, (n = 9, P = 0.041) although these results did not survive correction for multiple testing.

### Experiment 2: mating success

Previous experiments have shown population mean mating preferences for territorially successful males for previously mated females, but not virgins [18]. We wished to test whether females of different genotypes choose differently among males based on the male genotype and/or territorial status of the males. We tested three genotypes of females, all derived from the same Winters population as the male isogenic lines. These were similarly inbred, but of different genotypes from the males. A single female of one of the three genotypes was introduced into the Block 2 trials after scoring the males for territorial success. The male genotype that the female chose to mate with was recorded, as was his territorial status. Because mated females are known to have an overall preference for territorial males when remating, while there are conflicting results regarding virgin female territorial preferences [18], virgin females were utilised in order to maximise the chances of measuring preference differences between lines. While males might coerce mating, studies of Drosophila have consistently shown that females exercise considerable control over mating [20], and we took mating to indicate a measure of female preference.

#### Genetic variation in mating success

Disregarding male territorial success, only one of the female lines, 46, demonstrated significant mating discrimination among male lines overall (n = 135, df = 9: line 46, χ^2^ = 18.0, P = 0.036; line 137, χ^2^ = 6.6, P = 0.683; line 65, χ^2^ = 3.7, P = 0.928). Line 46 demonstrated significant preferences for 3 male lines (Table 2) in a series of χ^2^ tests against the neutral expectation, even when corrected for multiple testing. Neither of the other female lines demonstrated choice among male lines to a significant degree. In a χ^2^ test of differences among female lines for their choice of males, there was a significant difference in the way female lines 46 and 137 chose (n = 135, df = 9: 46×137 χ^2^ = 21.142, P = 0.012; 46×65 χ^2^ = 13.538, P = 0.140; 65×137 χ^2^ = 10.652, P = 0.300), but no differences were shown between 46 and 65, or 137 and 65.

**Table 2:** Relative mating success among inbred lines of *D. melanogaster*.

Male Line	Female Line Choosing			
	46*	65	137	Total
Agg	13	16	11	40
W145	**5** **	14	13	32
W75	18	13	10	41
W134	**22** **	15	16	**53** **
W17	11	9	11	31*
W58	**6** **	8*	16	30*
W89	14	16	14	44
W83	17	18	14	49
W23	14	14	13	41
Neu	15	12	17	44
per trial n	27	27	27	81

Scores shown are counts in dyadic mate choice trials.

* P<0.05

** P<0.01

Scores that maintain significance under a sequential Bonferroni correction for multiple testing are indicated in bold.

Examining male mating success among male lines without regard to female genotype, there was no overall evidence for a strong hierarchy of relative mating success based on male genotype (n = 405, df = 9, χ^2^ = 13.0 , P = 0.163). However, when male lines were evaluated individually among all female genotypes, three male lines show evidence of non-random mating success (Table 2). For at least one of these lines, male line 134 (n = 27, df = 1, χ^2^ = 7.72, P = 0.005), this survives a Bonferroni correction for multiple testing.

#### Interactions between male territorial success and mating success

Although some significant differences in mating success among male lines were obtained, there was no net effect of territorial success on mating success. A χ^2^ test of the effect of territorial success on mating success found no relation overall (n = 405, df = 9, χ^2^ = 8.79, P = 0.456), or for the different female lines analysed separately (n = 135, df = 9; line 46 χ^2^ = 8.99, P = 0.439; line 65 χ^2^ = 14.62, P = 0.102; line 137 χ^2^ = 11.19, P = 0.263). We also compared the relative number of times that males mated when holding a territory against the neutral hypothesis that males from a given line have an equal chance of mating when holding a territory or not. There was no indication that females chose differently between males on this basis, considering overall female mate choice (n = 405, df = 17, χ^2^ = 10.27, P = 0.329), or for any individual female line (n = 135, df = 17: line 46, χ^2^ = 9.11, P = 0.428; line 65, χ^2^ = 9.57, P = 0.386; line 137, χ^2^ = 9.20, P = 0.419).

While there was no overall relationship between territorial success and mating success, for particular male lines winning the territorial contest was a predictor of mating success (Table 3). Fisher's exact tests on the number of trials in which each of the 4 possible outcomes was obtained (mating, or not, after winning a territorial encounter, or not) showed that mating and winning a territory were related for individual male genotypes. For the Agg line, winning a territory increased mating success, while losing a territory was correlated with failure to mate, particularly with females from line 65 choosing (n = 27, P = 0.002) – this pattern is only borderline significant among females overall (n = 81, P = 0.06). For male line 134, there is also an indication that winning an aggressive encounter correlates with a greater ability to win mates (n = 81, P = 0.035). The opposite is true for males from line 27, a relatively non-aggressive line. Both when females from line 137 are choosing (n = 27, P = 0.006) and among all females overall (n = 81, P = 0.018), there is an apparent inverse relationship between winning an aggressive encounter and mating. While none of these p-values are significant under a Bonferroni correction, there are more significant tests than would be expected at random – 4 rather than 2 – from 40 trials with a significance threshold of P = 0.05, suggesting that several of the tests are likely to be true positives [21].

**Table 3:** Mating success in relation to territorial success and female genotype.

Male Line	Female Line 46		Female Line 65		Female Line137		Total	
	Scores	P =	Scores	P =	Scores	P =	Scores	P =
Agg	12, 10, 1, 4	0.326	**16, 5, 0, 6**	**0.002**	7, 13, 4, 3	0.391	35, 28, 5, 13	0.060
W145	5, 16, 0, 6	0.555	8, 4, 6, 9	0.252	11, 10, 2, 4	0.648	24, 30, 8, 19	0.234
W75	12, 4, 6, 5	0.411	8, 9, 5, 5	1.000	7, 11, 3, 6	1.000	27, 24, 14, 16	0.649
W134	14, 2, 8, 3	0.371	13, 7, 2, 5	0.185	9, 3, 7, 8	0.239	**36, 12, 17, 16**	**0.035**
W17	8, 6, 3, 10	0.120	4, 8, 5, 10	1.000	6, 8, 5, 8	1.000	18, 22, 13, 28	0.258
W58	1, 10, 5, 11	0.350	6, 8, 2, 11	0.209	5, 8, 11, 3	0.054	12, 26, 18, 25	0.366
W89	8, 5, 6, 8	0.450	8, 5, 8, 6	1.000	7, 4, 7, 9	0.440	23, 14, 21, 23	0.263
W83	6, 3, 11, 7	1.000	7, 3, 11, 6	1.000	3, 5, 11, 8	0.420	16, 11, 33, 21	1.000
W23	2, 6, 12, 7	0.103	4, 2, 10, 11	0.648	**2, 10, 11, 4**	**0.006**	**8, 18, 33, 22**	**0.018**
Neu	4, 1, 11, 11	0.342	3, 7, 9, 8	0.424	4, 2, 13, 8	1.000	11, 10, 33, 27	1.000

Scores shown for each combination of male and female genotype are, in order: mating success with territorial success; failure to mate with territorial success; mating success with territorial failure; and failure to mate with territorial failure.

Interactions that demonstrate a significant one-way relationship between territorial and mating success are highlighted in bold.

## Discussion

Influences of genotype on the outcome of behavioural interactions were found among lines of *D. melanogaster* for traits relevant to fitness – mating success and territorial success. Non-additive effects of genotype on behavioural outcomes were particularly strong for territorial interactions. Consistent with previous studies of territoriality and aggression in *D. melanogaster* [1], [15], [22], there were very large overall differences between males of different lines in their propensity to win territorial interactions. These relationships seemed entirely transitive among the Winters lines - which were isogenised immediately upon collection from nature, and represent a sample of the natural variation present in a single population.

High levels of intergenomic additivity for territorial success suggest that there is no strong directional selection for territorial behaviour in the population from which the Winters lines were sampled. If there were a direct relationship between territorial success and fitness, this variation would be expected to be depleted [7]. Some of this variation might be explained by environmental heterogeneity, or migration between populations in nature. But even in the constant environment of population cages, such as those used to maintain the stocks from which the Agg and Neut lines were derived, a great deal of genetic variation in aggressive behaviour can be maintained after many generations in the lab[1].

We found that not all the IGEs for territorial success are transitive between genotypes. The artificially selected Agg line represents an extreme phenotypes beyond that seen in any of the naturally derived lines. The Agg line for the most part prevailed in its aggressive interactions against all of the Winters lines. It was much less likely to hold a territory, however, when competed against one of these lines – line Winters 89, which otherwise was not notable for its level of pugnacity. Aggressive encounters in *D. melanogaster* might thus be susceptible to rock-scissors-paper dynamics similar to those found in other species [8]. The Agg line was selected to utilise a single, maximally aggressive strategy – tussling [1]. If such extreme phenotypes are commonly vulnerable to more moderate strategies, as we have shown, it may be one reason why populations do not evolve towards a uniformly maximally aggressive behavioural type. In such a system, even if territorial success confers an overall fitness advantage, no single aggressive strategy exists that can dominate all others, and go to fixation.

One way in which male territorial success could confer a fitness advantage is if territoriality helps to gain mating opportunities, either by coercion of females through holding a food and egg-laying resource, or by serving as a sexual signal in itself. An overall preference for males holding territories has in fact been found among mated *D. melanogaster* females in previous studies [18], suggesting there is likely to be some overall positive fitness effect to holding territory. Among virgin females, the relationship between territoriality and mating success is less consistent, and Hoffmann and Cacoyianni have shown population differences in virgin female preference for territorial males [18]. We utilised virgin females for our mate-choice tests, and similarly found no overall effect of territoriality on male mating success. Within our lines, rather, we found that the relationship between territorial success and mating success was specific to combinations of male and female genotypes, and did not find that male territorial success predicted mating success among females generally, or for any given genotype of females. While in the selected Agg line territorial success is a good predictor of male mating success, in one of the least aggressive lines, Winters 23, there is an inverse relation between mating success and territorial success. There are other patterns of mating success in our lines that show no relation with aggression. In most lines there is no association between territorial success and mating success, and three lines with intermediate territoriality have some of the highest, and lowest, levels of mating success overall.

In other animals, females have been shown to differ in their preferences for male sexual signals [10], and males have been shown to use different mating or display strategies in order to win mates [8]. Territorial success seems to act as a sexual signal in *D. melanogaster*, but male-male aggressive interactions do not define mating success. Females are evidently paying attention to other cues when making choices between combinations of males of different genotypes. Sexual signals like cuticular hydrocarbons and wing song are also known to be important for mate choice in Drosophila [23]. If winning and holding a territory is energetically expensive, some male genotypes might do better allocating their resources to expression of attractive CHCs or wing song – wooing rather than winning. Interactions between territoriality, signalling and female preference are known to maintain polymorphism in lizards [8], but have not been shown before in Drosophila.

### Concluding paragraph

Many theories for the maintenance of variation of behavioural traits have focussed on life history tradeoffs [24]–[26]. Sexual selection theory in the light of IGEs suggests that we might also look for interactions between genotypes to understand some of the genetic variation found in behavioural traits within populations [5], [7]. We found that interactions between genotypes were often not predictable from their population mean results, and we demonstrated intergenomic epistasis within a relatively small sample of naturally occurring and selected lines of *D. melanogaster*. The results of male-male aggressive interactions, while largely transitive, in some cases strongly depart from the expectation population-mean values leads us to expect, particularly for .the most aggressive phenotypes. Theory predicts that one outcome of directional selection for competitive fitness traits is the accumulation of nonlinear intergenomic interactions in the population [7], and we have shown that this is plausible. Directional selection, perhaps through female mate choice [18], on territorial success in males may thus be a diversifying force, given the presence of intransitive aggressive interactions between genotypes. Mating choices, though, can be contingent on genotypic and behavioural context in unexpected ways. Females of different genotypes choose differently among males, and while in some male genotypes, territorial success seems to help in acquiring mates, in others it detracts from mating success. In *D. melanogaster*, genetic variation in female mating preferences between genotypes, and across varying outcomes of interactions between males, may have implications for the maintenance of variation in territoriality and sexual signalling, even in apparently homogenous environments.

## Materials and Methods

### Fly lines and rearing

Eleven isogenic lines of *D. melanogaster,* collected from an orchard in Winters, California in 1998 were used in this experiment [27]. The Winters lines were made isogenic by 40 generations of full-sib breeding, and then maintained in mass culture. In addition, we studied two lines developed through a population-based selection procedure that increased aggression in one line (Agg), while no selection for increased aggression was applied to the second (Neut) line [1]. Flies were controlled for density and maintained under constant environmental conditions (12∶12 L∶D; 25°C) throughout the experiment. All flies, male and female, used in behavioural trials were virgin, and collected within a seven hour period after eclosion, anaesthetized with CO_2_ for sexing, and held singly in vials. Body size was not controlled because there is little evidence of an effect of body size on territorial success in *D. melanogaster* [28] .

### Experiment 1: aggression assays

Using a standard protocol for assessing aggression and territoriality in *D. melanogaster* [19], [29], an arena was constructed to provoke a zero-sum aggressive competition over a perceived high-quality territorial resource (yeast paste). A circular chamber was constructed by taping the bottom halves of two clear petri dishes (100 mm×20 mm) together. Within the chamber, a hexagonal weigh boat, 15 cm^2^×1 cm, of standard laboratory yeast-agar Drosophila medium was placed, with a small ball of baker's yeast paste (approximately 5 mm diameter) in the centre. The arena was encircled with cardboard to allow only light from above to enter. The temperature was maintained at 25°C. A small hole was drilled into the upper petri dish to allow introduction of flies into the arena.

Males from eight of the Winters isogenic lines were used in the aggression assays in two blocks (Blocks 1 and 2). Block 2 was conducted several months after Block 1, but the aggression aspect of the assay was performed in an identical manner. In addition to the Winters isogenic lines, the Agg and Neut lines were competed in the second of these blocks. Among the 8 lines utilised in Block 1 there are 28 unique between-line pairings, and each of these combinations were replicated 10 times each – for a total of 280 trials. Among the 10 male tester lines in Block 2, there are 45 possible non-redundant fighting combinations, and each of these unique pairings was replicated 9 times for a total of 405 paired aggression trials.

All rearing protocols were standard. Males were collected as virgins and aged individually and marked at 4 days of age with fluorescent powder. Half of these males were dusted with florescent powder, and all lines were aged a further 24 hours prior to fighting. Marking was randomised with respect to line, and had no statistical effect on the outcome of the mating or aggression trials (Block 1: n = 280, df = 1, χ^2^ = 0.000, P = 1.000, Block 2: n = 405, df = 1: aggression, χ^2^ = 0.71, P = 0.398; mating, χ^2^ = 0.56, P = 0.456 ). At subjective dawn on day 5 of the males' adult life, both males were transferred into the arena and at the end of 24 hours, all dyads in both blocks were observed for an hour, and all were found to have an unambiguous winner – a single male occupied the food area, and chased off the other male if intrusion occurred. In no trials was a change in territorial occupant noted in an hour of observation.

The significance of differences among the lines for territorial success was tested with a χ^2^ test, with a null expectation of an equal number of wins for each genotype. Individual lines were also tested for their departure from population mean success with a null expectation of an equal numbers of wins to losses. While these tests indicate the significance of differences in aggression levels among genotypes, we also tested whether these overall results (against the population mean expectation) correlate well with the realised results of the pairwise interactions between male genotypes.

For each pairwise interaction, two-by-two contingency tables were constructed, indicating the number of wins of the focal genotype and those of the opponent genotype (which represent interaction-specific measures of territorial success). The null expectation was the number of wins of the focal and opponent genotypes in all trials *excluding* those being tested. The null thus represents a measure of population-level territorial success for each genotype, and is proportional to the expected number of wins for each genotype. Due to the small number of results in some of the cells of the contingency tables (<5), χ^2^ tests could not be used, so Fisher's exact tests were used to determine departure from the expected number of wins to losses (Table 1).

### Experiment 2: mate-choice protocol

Females from three Winters inbred lines were used to assess relative male mating success in the context of the territorial assay in Block 2. A single virgin female from one of three Winters lines, collected and aged in the same way as the males, was introduced into each of the territorial arenas following scoring for territorial success. Each female was placed in a small tube, from which she was allowed to enter the arena at her discretion, and the identity of the first male she mated with was then recorded. All females mated within 2 hours. The nine replicates of the 45 combinations of males assessed in the territorial assay were assessed by three individual females from each of the three Winters assessing lines, randomly assigned.

The significance of several kinds of genotype×genotype interactions were assessed from the results of these trials. Temporarily ignoring the outcome of aggressive encounters, the differences in relative male mating success were assessed by χ^2^ testing at both the female level (whether females demonstrate choice overall among male lines), and at the level of individual male genotype, given the female choosing (Table 2). Overall variation in choice among genotypes, and for particular male genotypes, was also assessed for all female genotypes pooled. Pairwise comparisons between the mating choices of each of the female genotypes were examined, and χ^2^ testing used to see if there are genetic differences among females in their mating choices. Interactions between territorial success, male genotype and female genotype on mating success were tested using Fisher's exact test, assuming no effect of territorial success on mating success (Table 3).
